# Oleic, Linoleic and Linolenic Acids Increase ROS Production by Fibroblasts via NADPH Oxidase Activation

**DOI:** 10.1371/journal.pone.0058626

**Published:** 2013-04-08

**Authors:** Elaine Hatanaka, Alexandre Dermargos, Aparecida Emiko Hirata, Marco Aurélio Ramirez Vinolo, Angelo Rafael Carpinelli, Philip Newsholme, Hugo Aguirre Armelin, Rui Curi

**Affiliations:** 1 Institute of Physical Activity and Sport Sciences, Cruzeiro do Sul University, São Paulo, Brazil; 2 Department of Physiology and Biophysics, Institute of Biomedical Sciences, University of São Paulo, São Paulo, Brazil; 3 Instituto de Química, Universidade de São Paulo, São Paulo, Brazil; 4 Laboratório Especial de Ciclo Celular, Instituto Butantan, São Paulo, Brazil; 5 Departamento de Fisiologia, Universidade Federal de São Paulo, São Paulo, Brazil; 6 Department of Genetics, Evolution and Bioagents, Institute of Biology, University of Campinas, Sao Paulo, Brazil; 7 School of Biomolecular and Biomedical Science, Conway Institute, University College Dublin, Dublin, Ireland; 8 School of Biomedical Sciences, Curtin University, Perth, Western Australia; Laurentian University, Canada

## Abstract

The effect of oleic, linoleic and γ-linolenic acids on ROS production by 3T3 Swiss and Rat 1 fibroblasts was investigated. Using lucigenin-amplified chemiluminescence, a dose-dependent increase in extracellular superoxide levels was observed during the treatment of fibroblasts with oleic, linoleic and γ-linolenic acids. ROS production was dependent on the addition of β-NADH or NADPH to the medium. Diphenyleneiodonium inhibited the effect of oleic, linoleic and γ-linolenic acids on fibroblast superoxide release by 79%, 92% and 82%, respectively. Increased levels of p47*^phox^* phosphorylation due to fatty acid treatment were detected by Western blotting analyses of fibroblast proteins. Increased p47*^phox^* mRNA expression was observed using real-time PCR. The rank order for the fatty acid stimulation of the fibroblast oxidative burst was as follows: γ-linolenic > linoleic > oleic. In conclusion, oleic, linoleic and γ-linolenic acids stimulated ROS production via activation of the NADPH oxidase enzyme complex in fibroblasts.

## Introduction

Plasma fatty acid levels are elevated during conditions such as diabetes and inflammation, which are commonly associated with ROS production and the development of excess fibrous connective tissue in organs or tissues due to fibroblasts [1], [2], [3], [4].

ROS modulate the activity of signalling pathways involved in fibroblast migration, proliferation, connective tissue deposition, vascular tone and senescence [5], [6]. ROS play a role in the activation of NF-κB, phospholipase D, protein kinase C (PKC) and mitogen-activated protein kinase (MAPK) in response to various agonists [5], [7]. ROS are also required for the activation of Akt, p70S6K and G-proteins, such as Gi, Go, and for the expression of early growth response factor-1 (EGR) and vascular endothelial growth factor (VEGF). The activation of signalling cascades by low levels of ROS results in increased cell cycle progression. For example, the proliferative state of fibroblasts is tightly associated with intracellular ROS levels. Low ROS levels lead to cell growth arrest, which is induced by contact inhibition. On the other hand, the overproduction or insufficient scavenging of ROS can result in enhanced fibrosis and oxidative stress, which have been implicated in several diseases [2], [8], [9]. The regulation of the intracellular redox state by fatty acid-induced changes in NAD(P)H oxidase activity is thought to have an important impact on redox-sensitive signalling cascades.

NADPH oxidase was originally believed to be present in phagocytes only; however, its expression has been demonstrated in several non-phagocytic cell types such as vascular smooth muscle cells [10], pancreatic β cells [11] and fibroblasts [12], [13]. ROS are produced by NADPH oxidase homologues (known as NOX) in non-phagocytic cells. The NADPH oxidase subunits gp91*^phox^* and p22*^phox^* are integral membrane proteins [2]. These subunits form heterodimeric flavocytochrome b558, which forms the catalytic core of the enzyme. However, this enzyme exists in an inactive state in the absence of the other subunits. Additional subunits are required for regulation and are located in the cytosol during the resting state. These subunits include the proteins p67*^phox^*, p47*^phox^* and p40*^phox^* and the small GTPase Rac. p47*^phox^* is phosphorylated at multiple sites by a number of protein kinases, including members of the PKC family, and it is important in the regulation of NADPH oxidase activity. Cell stimulation leads to the phosphorylation and translocation of p47*^phox^* to the membrane. In the membrane, p47*^phox^* and p67*^phox^* directly interact with and activate NOX [2]. gp91*^phox^*, p67*^phox^* and p47*^phox^* expression has been reported in fibroblasts from different species [12], [13].

Oleic (C18∶1), linoleic (C18∶2) and γ-linolenic (C18∶3) acids are abundant fatty acids in human and rat plasma [14]. These fatty acids have the same carbon atom chain length but different degrees of unsaturation and positions of the double bonds in their molecules. Although fatty acids have been demonstrated to activate NADPH oxidase in leukocytes [15] and pancreatic β cells [16], the effect of oleic, linoleic and γ-linolenic acids on NADPH oxidase in fibroblasts has not yet been investigated. Given that fibroblasts exert profound effects on the progression of inflammatory chronic diseases, the goal of the present study was to investigate the effect of fatty acids on intracellular and extracellular ROS levels in cultured fibroblasts (the 3T3 Swiss and Rat 1 cell lines) using three techniques: lucigenin-amplified chemiluminescence, reduction of hydroethidine and phenol red reduction. The levels of p47*^phox^* phosphorylation and p47*^phox^* mRNA expression in response to the addition of oleic, linoleic and γ-linolenic acids were detected using Western blot analysis and real-time PCR, respectively.

## Materials and Methods

Dulbecco's modified Eagle's medium, HEPES, ampicillin, streptomycin, Trizol reagent, random pd(N)_6_ primers, DNAse I, Superscript II RT and Taq DNA polymerase were purchased from Invitrogen Life Technologies (Carlsbad, CA, USA). Fatty acids, lucigenin, hydroethidine, hydrogen peroxide, β-NADH, NADPH, and phenol red were supplied by Sigma Chemical Co. (St. Louis, MO, USA). The p47phox rabbit antibody was obtained from Santa Cruz Biotechnology (Santa Cruz, CA, USA). The fatty acids were dissolved in ethanol. The final concentration of ethanol in the assay medium did not exceed 0.05%. At this concentration, a preliminary experiment demonstrated that ethanol is not toxic to fibroblasts and that it does not interfere with the obtained results. Reagents, water and plastic wares used in the experiments were all endotoxin free.

### 2.1. Preparation and treatment of the fibroblasts

Swiss 3T3 and Rat 1 fibroblasts, which were obtained from the American Type Culture Collection, were maintained at 37°C in Dulbecco's modified Eagle's medium supplemented with 25 mM HEPES (pH 7.4), 10% (v:v) foetal bovine serum, 25 μg per mL ampicillin, and 100 μg per mL streptomycin (culture medium). For all experiments, cells were used between passages 5 and 7. When the cells reached confluency, adherent fibroblasts were treated with trypsin, gently washed, and resuspended in PBS buffer. The cells were maintained on ice until they were assayed. For the chemiluminescence assay, 2.5×10^6^ cells per mL were added to 0.3 mL of pre-warmed (37°C) PBS buffer containing lucigenin (1 mM), phenol red (0.28 mM) or hydroethidine (1 µM). The cells were treated with various concentrations (0, 5, 25, 50, 100, or 200 μM) of oleic, linoleic or γ-linolenic acids. ROS release was monitored for 30 min after the addition of the fatty acids. The assays were performed in PBS buffer supplemented with CaCl_2_ (1 mM), MgCl_2_ (1.5 mM), and glucose (10 mM) at 37°C in a final volume of 0.3 mL.

### 2.2. Cell viability

The treated cells were centrifuged at 1,000× g for 15 min (4°C), and the resultant pellet was resuspended in 500 μL phosphate-buffered saline (PBS). Afterwards, 50 μL of propidium iodide solution (50 mg/mL in PBS) was added, and the cell viability was analysed using a FACSCalibur flow cytometer (Becton Dickinson, San Juan, CA, USA). Propidium iodide is a highly water-soluble fluorescent compound that cannot pass through intact membranes, is generally excluded from viable cells and binds to DNA by intercalating between the bases. Fluorescence was measured using the FL2 channel (detection from 564–606 nm). Ten thousand events were analysed per experiment. Cells with propidium iodide fluorescence were then evaluated using the Cell Quest software (Becton Dickinson, San Juan, CA, USA). Viability was always greater than 98.5%, as indicated by flow cytometry.

### 2.3. Lucigenin-enhanced chemiluminescence assay

Lucigenin is extensively used to measure the production of reactive oxygen species by chemiluminescence [15]. After being excited by the superoxide anion, lucigenin releases energy in the form of light. Lucigenin-amplified chemiluminescence is a specific method for studying the kinetics of the superoxide production of cells. Using this method, the response to xanthine/xanthine oxidase results in a positive correlation with light measurements; however, this method does not indicate augmented chemiluminescence when peroxidase is added to the assay medium [17]. In addition, chemiluminescence is inhibited in a dose-dependent manner by scavengers of superoxide anions, e.g., SOD, but not by azide, catalase, mannitol or taurine. Thus, this is a specific method to measure superoxide anion production [18]. The chemiluminescence response was monitored for 30 min (37°C) using a microplate luminometer (model LB96V, EG&G Berthold; Bad Wildbad, Germany).

### 2.4. Flow cytometric measurements of reactive oxygen metabolites using hydroethidine

Hydroethidine (HE) has been widely used for the flow cytometric measurement of intracellular ROS levels. Hydroethidine, a reduced derivative of ethidium bromide, easily penetrates into cells and displays weak fluorescence when excited by light (480 nm). Hydroethidine is oxidised intracellularly by oxygen radicals and converted into ethidium bromide, which tightly binds to DNA and displays a strong red fluorescence [19]. One advantage of this method is the ability to evaluate the response of individual cells. Fibroblasts were treated as described above in the presence of hydroethidine (1 µM). Fluorescence was measured using the FL3 channel (detection above 670 nm) in a FACSCalibur flow cytometer (Becton Dickinson, San Juan, CA). Ten thousand events were analysed per experiment.

### 2.5. Hydrogen peroxide determination

For measuring hydrogen peroxide levels, we used a single, rapid and inexpensive method described by E. Pick et al. [20]. This assay is based on the horseradish peroxidase (HRP)-mediated oxidation of phenol red by H_2_O_2_. The reaction leads to the formation of a coloured compound whose maximum absorbance occurs at 610 nm [20]. The phenol red assay allows for the detection of reactive oxygen species both inside and outside of cells. Fibroblasts were treated as described above in the presence of phenol red (0.28 mM) and 1 U/mL peroxidase (horseradish Type II, (HRP)). The reaction was stopped by the addition of 10 µL of a 1 N sodium hydroxide aqueous solution.

### 2.6. Western blot analysis of p47phox phosphorylation

In the Western blot experiments, the total protein levels of each sample were quantified by the Bradford method. After this procedure, the same amount of protein was immunoprecipitated using the anti-phosphoserine antibody. After the immunoprecipitation, the samples were immunoblotted using anti-p47phox antibody. After incubating the cells at 37°C in the absence (0 min) or presence of fatty acids (100 µM – for 5, 10 or 15 min), they were homogenized in extraction buffer and prepared for Western blot analysis.

The cells were homogenised in extraction buffer (100 mM Tris (pH 7.5), 10 mM EDTA, 100 mM NaF, 10 mM sodium pyrophosphate, 10 mM sodium orthovanadate, 2 mM phenylmethanesulfonyl fluoride, 0.01 mg/mL aprotinin) at 4°C for 30 s. After homogenisation, Triton X 100 was added to 1%, and the samples were incubated for 30 min (4°C). Samples were centrifuged at 13,000× *g* for 20 min (4°C). Each sample (250 µg) was immunoprecipitated using the anti-phosphoserine antibody (1∶300 dilution). Immunoprecipitated samples were mixed with protein A-Sepharose for 4 h (4°C), subjected to electrophoresis and then immunoblotted using an anti-phosphoserine antibody. Briefly, the gel was transferred to a nitrocellulose membrane at 120 V for 1 h. The membrane was blocked with 5% defatted milk in a basal solution (10 mM Tris (pH 7.5), 150 mM NaCl, 0.05% Tween 20) at room temperature for 2 h. The membranes were washed 3 times (10 min for each wash) in basal solution and then incubated at room temperature for 3 h with an anti-p47phox antibody (1∶3,000 dilution) in a basal solution containing 3% defatted milk. The membranes were washed again (3 washes, 10 min each) and incubated with an anti-IgG antibody (1∶10,000 dilution) linked to horseradish peroxidase in a basal solution containing 1% defatted milk at room temperature for 1 h. Following the washings, the membranes were incubated with the substrate for peroxidase and a chemiluminescence enhancer (ECL Western Blotting System Kit, GE Health Care, Little Chalfont, Buckinghamshire, England) for 1 min and immediately exposed to X-ray film. The films were then developed. The band intensities were quantified using optical densitometry.

### 2.7. Real-Time Reverse Transcriptase Chain Reaction-PCR

After being incubated at 37°C for 1 hour in the absence or presence of fatty acids (100 µM), RNA was obtained from Rat 1 fibroblasts using the Trizol reagent. The expression of NADPH oxidase component p47^phox^ was evaluated using real-time PCR in a Rotor Gene 3000 PCR machine (Corbett Research, Mortlake, Australia) using the method described by Higuchi *et al*. (1992) [21]. cDNA probes were synthesised using 4 μg of total RNA and a mixture of the following reagents: 146 ng of “random primers”, 200 U of Superscript reverse transcriptase, 5× reaction buffer (50 mM Tris – HCl (pH 8.0), 75 mM KCl, 3 mM MgCl_2_), 5 mM DTT, and 500 μM dNTP in a final volume of 20 μL. The reaction was incubated for 2 min (25°C) to assemble the oligonucleotides and hybridise the RNA, which was followed by heating at 42°C for 50 min. The cDNA was stored at −20°C prior to the real-time PCR assay. To perform the real-time PCR reaction, 1 μL of cDNA was used in a final volume of 25 μL, which contained 100 μM of dNTPs, 10× reaction buffer (10 mM Tris–HCl, 50 mM KCl, 2 mM MgCl_2_), 1 U of Taq DNA polymerase, 0.1 μM of each primer (sense and antisense), and SYBR GREEN (1000× diluted) as the fluorescent dye. The primer sequences were designed using information from GenBank (National Center for Biotechnology Information (NCBI)). The quantification of gene expression was performed using the method described by Liu and Saint (2002) and the β2 myosin gene as an internal control [22].

### 2.8. Statistical analyses

Comparisons were performed using a one-way ANOVA and Dunnett's Test. The significance was set at p<0.05. The results were obtained from 3 to 5 independent experiments and are expressed as the mean ± SEM.

## Results

To assess any possible interference of the fatty acids with the ROS reaction involving the reagents, the appropriate controls were performed by using 10, 25, 50, 100, and 200 μM oleic, linoleic or γ-linolenic acids in the assays (lucigenin and phenol red) without cells. The three fatty acids did not directly affect the lucigenin and phenol red assays, as reported in our previous study [15]. Controls using the vehicle (ethanol) for the fatty acids were also provided.

**Figure 1:**
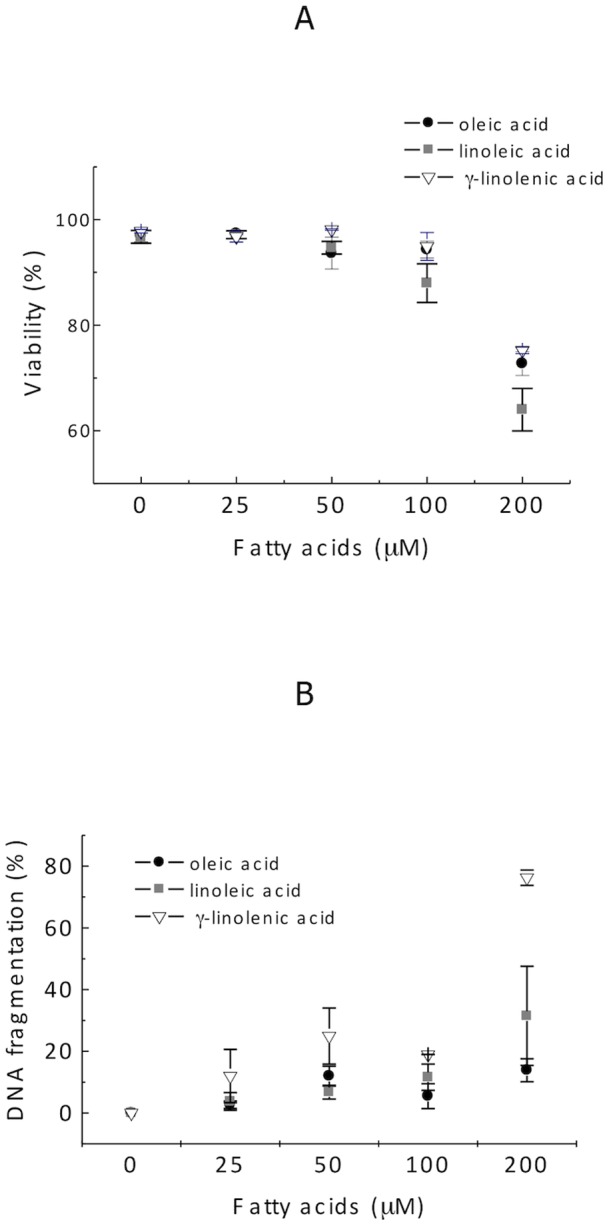
Analysis of membrane integrity and DNA fragmentation. Confluent 3T3 Swiss fibroblasts were treated with vehicle control (0) or oleic, linoleic or γ-linolenic acid (10, 20, 50, 100 or 200 μM). The results are presented as the mean ± standard error of at least three experiments that were performed in duplicate.

In order to verify the influence of fatty acids on fibroblast viability, flow cytometric analysis was used to determine cell membrane integrity and DNA fragmentation of fibroblasts treated with oleic, linoleic and γ-linolenic acid (25, 50, 100 and 200 µM) for 30 minutes. No significant effect of treatments were found at experimental condition of this study (Figure 1).

**Figure 2:**
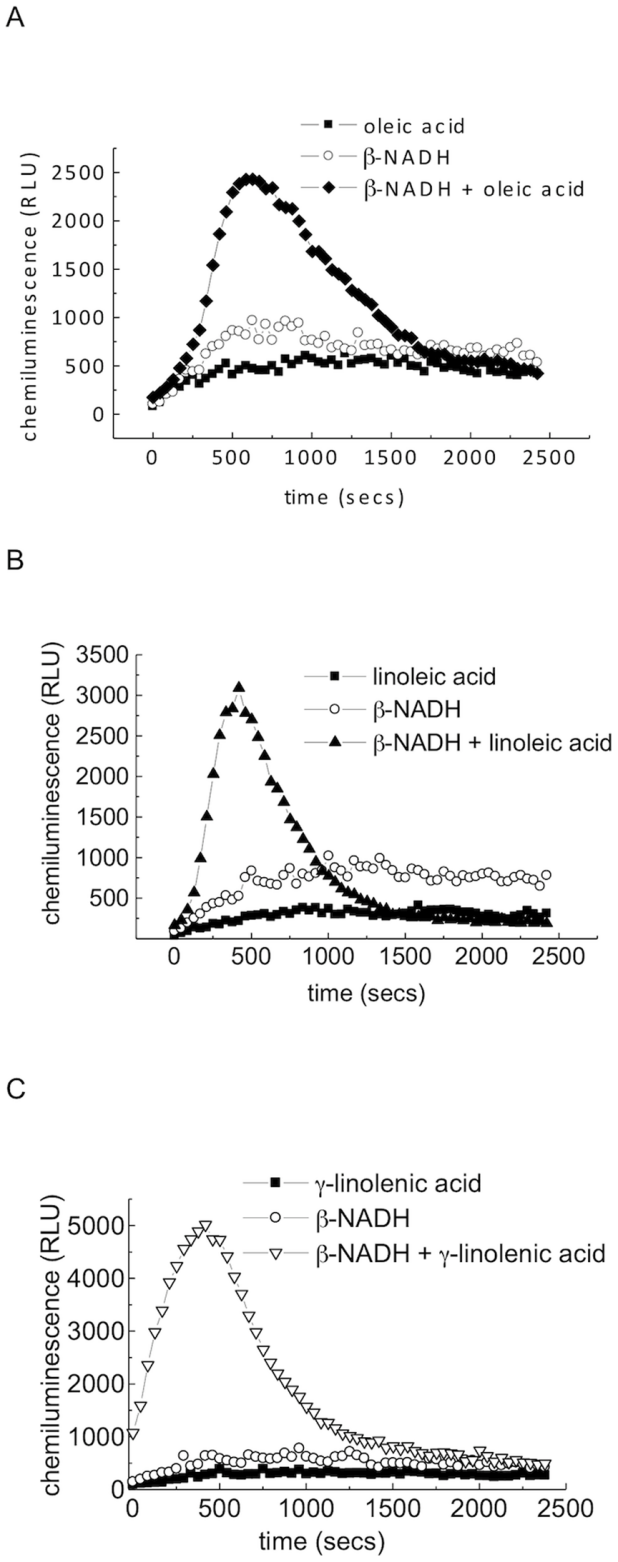
Comparative effect of fatty acids on the kinetics of superoxide production by fibroblasts. Cells (2.5×10^6^ cells per mL) were treated with oleic, linoleic or γ-linolenic acid (100 μM) plus β-NADH (10 µM) in the presence of lucigenin (1 mM).

In the presence of β-NADH (10 µM), a substrate for the oxidases [23], [24], the oleic, linoleic and γ-linolenic acids increased the extracellular superoxide anion (O_2_^•−^) level, as indicated by the lucigenin-amplified chemiluminescence assay (Figure 2). Significant superoxide levels were immediately detected after the fatty acid treatments and remained elevated for at least 20 min in the treated fibroblasts. In the absence of β-NADH or NADPH, there was no chemiluminescence above the background level. The kinetic studies indicated that the induction of superoxide production in the fibroblasts is a fast event that occurs within minutes after treatments with the oleic, linoleic and γ-linolenic acids (Figure 2). Our results demonstrate that the three fatty acids increased fibroblast oxidative bursts as follows: γ-linolenic > linoleic > oleic. The period for maximal superoxide production was 8.2±2.6 min for oleic acid, 3.8±1.6 min for linoleic, and 3.1±1.2 min for γ-linolenic. An interesting aspect is that as the number of double bonds in the 18-carbon fatty acid molecule increased, so did its ability to stimulate oxidative bursts in the 3T3 Swiss and Rat 1 fibroblasts.

**Figure 3:**
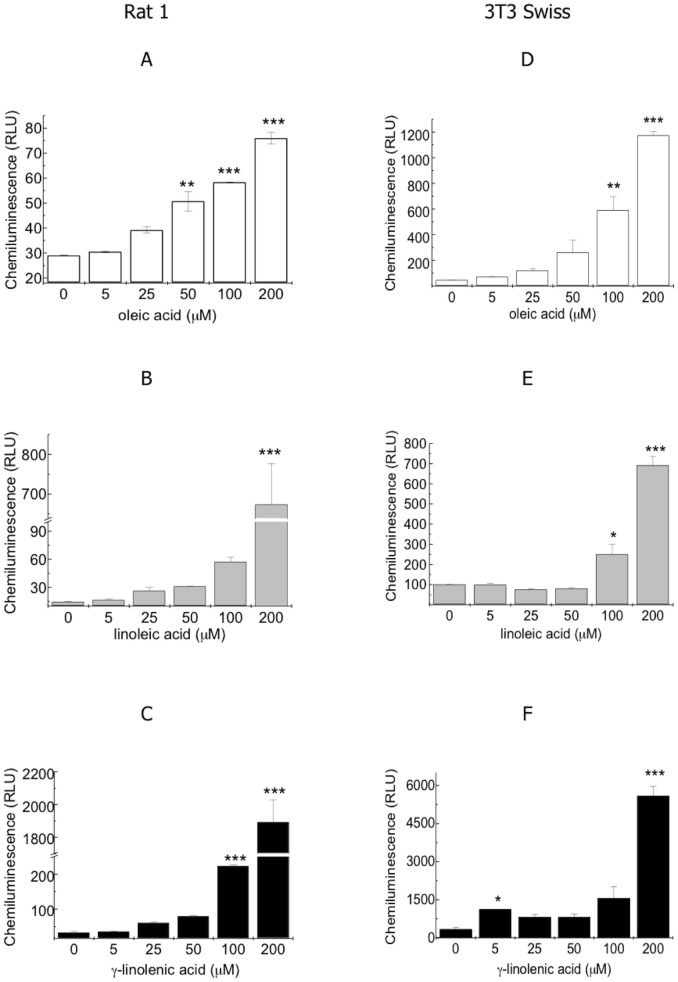
Dose response treatment with the fatty acids. Superoxide anion levels in the incubation media for the Rat 1 (A, B and C) and 3T3 Swiss fibroblasts (D, E and F) (2.5×10^6^ cells per mL) containing β-NADH (10 µM), as measured by the lucigenin assay in the absence and presence of different concentrations of oleic (A and D), linoleic (B and E) or γ-linolenic (C and F) acid (0, 5, 50, 100 or 200 μM). The results are presented as the mean ± SEM of at least three experiments performed in triplicate. Presented as the effect of the fatty acids compared with the control, *p<0.05, ** p<0.01 and *** p<0.001.

There was a positive correlation between fatty acid concentration (zero, 5, 25, 50, 100, or 200 μM) and superoxide production (Figure 3). The Pearson correlation coefficient determined for the Rat 1 fibroblasts was: r = 0.97 and p = 0.001 for oleic acid, r = 0.97 and p = 0.0008 for linoleic acid and r = 0.89 and p = 0.015 for γ-linolenic acid. The Pearson correlation coefficient for the Swiss 3T3 fibroblasts was: r = 0.63 and p = 0.1 for oleic acid, r = 0.75 and p = 0.08 for linoleic acid and r = 0.49 and p = 0.3 for γ-linolenic acid. No detectable superoxide production occurred when the fibroblasts were treated for 10 min with oleic, linoleic or γ-linolenic acid without the addition of β-NADH.

The addition of SOD (150 U/mL), a scavenger of the superoxide anion, to the assay system partially inhibited the chemiluminescence induced by the fatty acids. The responses of the 3T3 Swiss and Rat 1 fibroblasts were similar. The SOD control was performed by using inactive SOD (a heat-killed preparation), and the chemiluminescence induced by the fatty acids was not modified (data not shown).

**Figure 4:**
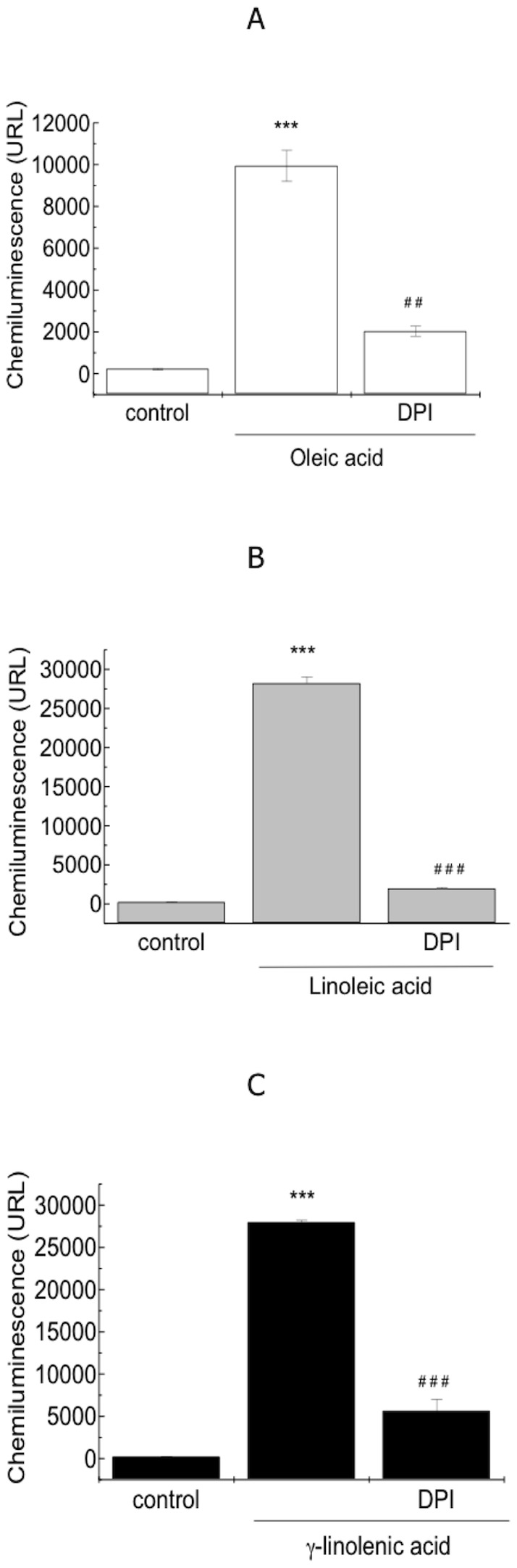
The involvement of NADPH oxidase in fatty acid-induced ROS production. Effect of DPI (200 µM), an inhibitor of NADPH, on the chemiluminescence of fatty acid-treated (100 µM) 3T3 Swiss fibroblasts. Presented as the effect of the fatty acids compared with the control, *p<0.05, ** p<0.01 and *** p<0.001.

The effect of the oleic, linoleic and γ-linolenic acids on fibroblast superoxide production was severely attenuated, i.e., reductions of 79%, 92% and 82%, respectively, when the cells were pre-treated for 5 min with diphenyleneiodonium (DPI), a specific inhibitor of NADPH oxidase (Figure 4).

**Figure 5:**
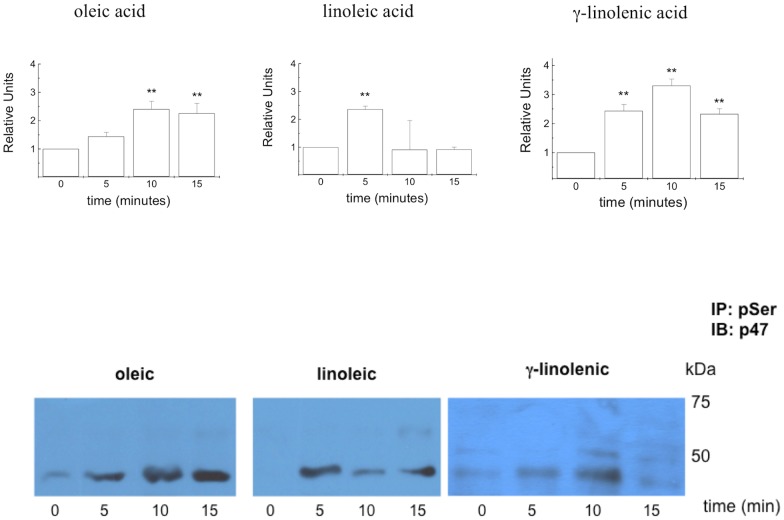
Phosphorylation of NADPH oxidase component p47^phox^ by fatty acids. Effect of oleic, linoleic and γ-linolenic acids on the phosphorylation of the NADPH oxidase component p47^phox^ in Rat 1 fibroblasts. After incubating the cells at 37°C in the absence (0 min) or presence of fatty acids (100 µM – for 5, 10 or 15 min), they were homogenized in extraction buffer and prepared for Western blot analysis. Western blotting was performed using a rabbit anti-p47^phox^ antibody. Similar results were obtained from three to four independent experiments. Presented as the effect of the fatty acids compared with the control, *p<0.05, ** p<0.01 and *** p<0.001.

The effect of the oleic, linoleic and γ-linolenic acids on the phosphorylation of p47^phox^ (an active catalytic subunit of NADPH oxidase) was investigated using Western blot analysis. Because the phosphorylation of p47^phox^ is a rapid event, the activation of p47^phox^ was analysed after 5, 10 and 15 min in Rat 1 fibroblasts incubated in the presence of oleic, linoleic or γ-linolenic acid (Figure 5). An increase in p47^phox^ mRNA expression following fatty acid treatment was observed by real-time PCR (Table 1).

**Table 1:** Effect of oleic, linoleic and γ-linolenic acids on the mRNA expression of NADPH oxidase component p47^phox^ in Rat 1 fibroblasts.

	p47^phox^ mRNA (relative units)
	(mean ± SD)
Control	1±0,04
Oleic acid	1766±176***
Linoleic acid	318±31***
γ-Linolenic acid	408±314***

Presented as the effect of the fatty acids compared with the control, *p<0.05, ** p<0.01 and *** p<0.001.

The treatment of fibroblasts with oleic, linoleic or γ-linolenic acid did not significantly increase intracellular levels of ROS, as indicated by hydroethidine reduction. In addition, these fatty acids did not significantly increase the basal levels of H_2_O_2_ in the 3T3 Swiss and Rat 1 fibroblasts, as indicated by the phenol red reduction assay under the conditions described herein (data not shown).

## Discussion

In this work, evidence is presented that oleic, linoleic and γ-linolenic acids increase extracellular superoxide levels in fibroblast cell lines through the activation of p47^phox^ and the stimulation of NADPH oxidase. We also observed that oleic, linoleic and γ-linolenic acids increase p47^phox^ mRNA expression, suggesting that these fatty acids induce the synthesis of this regulatory subunit.

Our results demonstrate that the tested fatty acids increased the fibroblast oxidative burst as follows: γ-linolenic > linoleic > oleic acids. Studies involving fatty acid structure and function have demonstrated that, in general, as the number of double bonds in the fatty acid molecule increases, so does its ability to stimulate oxidative bursts in unstimulated neutrophils [15]. According to Hardy (1994), exogenous long chain and very long chain fatty acids use the same signal transduction pathways to stimulate ROS production by neutrophils [25].

Our results suggest that NOX and p47phox are implicated in ROS production by fatty acids treated fibroblasts; however in the cases of oleic acid and γ-linoleic acid, the inhibition is incomplete, suggesting that additional mechanisms may be at work. Superoxide can also be generated through the mitochondrial electron transport chain, xanthine – xanthine oxidase and cytochrome P450. Mitochondria generate superoxide mostly through the univalent reduction of oxygen in complexes I and III of the electron transport chain.

NADH cannot cross biological membranes under normal conditions. The cytosolic concentration of β-NADH is 270 µM, whereas the mitochondrial concentration is 638 µM [26]. If tissue damage occurs, for instance during periods of prolonged ischaemia and/or cellular death due to necrosis, a loss of plasma membrane integrity is observed [26]. Under these conditions, fibroblasts are able to produce ROS in response to fatty acids. These conditions are present during acute inflammation and in the skeletal muscle of athletes during high intensity exercises, such as the marathon and triathlon. We have demonstrated that pre-treating 3T3 Swiss and Rat 1 fibroblasts for 5–10 min with oleic, linoleic or γ-linolenic acid enhances fibroblast ROS production when compared with a β-NADH or NADPH treatment only.

The increased superoxide production observed when fibroblasts are treated with fatty acids plus β-NADH or NADPH may indicate an effect of metabolites as priming agents. Classical priming agents alone are not able to stimulate cell oxygen consumption and ROS production but lead to an incremental increase in the maximal rate of oxygen consumption when a second stimulus occurs [27], [28]. Hardy et al. (1994) demonstrated that pre-treating neutrophils with arachidonic, eicosapentaenoic or docosahexaenoic acids enhances their ability to respond to either fMLP or PMA, thereby producing more superoxide than when challenged with the stimulators only [25]. Recently, we demonstrated that the addition of PMA to an assay medium leads to an additive effect on the superoxide and hydrogen peroxide production induced by oleic, linoleic and γ-linolenic acids in neutrophils [15].

In infectious/inflammatory processes, the source of fatty acids may be blood, extracellular fluids, bacterial cell membranes or infiltrating leukocytes. Under these conditions, levels of fatty acids and metabolites are increased, as are the number of dying cells, and hence, the β-NADH levels. In these situations, fatty acids released into the microenvironment can be an important mediator for the process of the resolution/progression of the damaged tissue through increased ROS production.

The high concentrations of fatty acids associated with the increased microvascular permeability observed in some diseases [29] may define specific loci, such as the interstitial space and body cavities, for fatty acid action. At these sites, fatty acids may activate neutrophils and macrophages and promote fibroblast ROS release. Fibroblast proliferation and fibrogenesis are important factors that lead to the complications of various diseases, such as atherosclerosis, rheumatic arthritis, diabetic nephropathy and retinopathy [30]. The data from this study indicate that oleic, linoleic and γ-linolenic acids are important inducers of ROS production by fibroblasts. This effect may be critical during certain physiological processes, such as wound healing. However, this effect could also be deleterious in proliferative diseases that involve fibroblast dysfunction, such as fibrosis, and by promoting uncontrolled inflammation [31]. These outcomes are more relevant in pathological conditions involving persistent increases in fatty acid serum levels, such as diabetes [32].

O'Donnell et al. (1996) demonstrated that fibroblasts treated with arachidonic, linoleic or (5S)-hydroxyeicosatetraenoic acid [(5S)-HETE] present enhanced superoxide generation. Their study suggests the involvement of 15-lipoxygenase on ROS production [26]. Maziere et al. (1999) reported that treating cultured human fibroblasts with oleic, linoleic or γ-linolenic acid increases their intracellular levels of ROS and lipid peroxidation products. This group also demonstrated the activation of the oxidative stress-responsive transcription factors AP1 and NF-κB [33].

Several studies have demonstrated the presence of NADPH oxidases in the homogenates and particulate fractions of endothelial and smooth muscle cells and that these NADPH oxidases are able to generate superoxide or hydrogen peroxide. Here, evidence has been presented that oleic, linoleic and γ-linolenic acids are potent inducers of ROS production in fibroblasts. These fatty acids stimulate ROS production via the dose-dependent activation of the NADPH oxidase complex. Excessive ROS production can damage cellular lipids, proteins and DNA, which impairs cell function. In fact, oxidative stress has been implicated in a number of human diseases and in the ageing process.

An increasing body of evidence indicates that in diseases such as metabolic syndrome [34], sepsis [35] and diabetes [36], the plasmatic concentrations of free fatty acids and ROS are higher. Considering the findings of the present study, we hypothesize that fatty acids, through their effects on fibroblasts, may contribute to the pro-oxidant state observed in these pathological conditions. Additionally, taking into account the fact that fibroblasts are essential cells in wound healing, a process that is impaired in diabetic individuals, fatty acids-induced production of ROS by fibroblasts may, together with changes in other cell types, be important in this context. In fact, ROS can activate neutrophils/macrophages to produce pro-inflammatory cytokines via NF-κB activation, and the pro-inflammatory status may contribute to chronic inflammation in non-healing wounds or in insulin resistance.
